# Clinical, biomechanical and histological study on oophorectomy induced menopause

**DOI:** 10.1590/1413-78522014220500420

**Published:** 2014

**Authors:** Maristela Bordinhon, Sérgio Swain Müller, Maeli Dal Pai Silva

**Affiliations:** 1 Faculdades Adamantinenses Integradas, Adamantina, SP, Brazil, Faculdades Adamantinenses Integradas, Adamantina, SP, Brazil; 2 Universidade Estadual Paulista, Faculdade de Medicina de Botucatu, Botucatu, SP, Brazil, Faculdade de Medicina de Botucatu da Universidade Estadual Paulista, Botucatu, SP, Brazil

**Keywords:** Ovariectomy, Rats, Female, Biomechanics, Histology, Osteoporosis, postmenopausal

## Abstract

**Objective:**

To investigate the clinical implications as well as biomechanical and histological changes and in bone tissue induced by ovariectomy in 64 rats.

**Methods:**

The rats were divided into two groups: bilateral oophorectomy or placebo, and subdivided into four subgroups, according to time postoperatively: three, six, nine and 12 months. The weight of the animals at the time of sacrifice was taken into consideration. The biomechanical study was performed on the right tibia, to the maximum load and stiffness coefficient. For the histological study we calculated the trabecular bone of the left tibia. Statistical analysis of body weight and mechanical properties was performed by variance analysis, complemented with Tukey's multiple comparison tests; and trabecular area, the non-parametric variance analysis.

**Results:**

Ovariectomy-induced menopause caused an increase in body weight, reduction of diaphyseal bone resistance at six months of hormone deprivation, but this effect is equalized over time by aging; bone stiffness was smaller in the ovariectomized group and reduction of bone mass occurred.

**Conclusion:**

The removal of the ovaries produced systemic alterations, characterized by metabolic changes that caused weight gain and changes in bone tissue, associated with alteration of the mechanical profile and reduced bone mass.* Level of Evidence I, Clinical Study.*

## INTRODUCTION

Osteoporosis can be defined as diffuse skeletal disease characterized histopathologically by an absolute decrease in bone mineralization. Osteoporosis is often a silent disease, until the occurrence of fracture. When compared to normal bone, osteoporotic bone shows reduction in the number and thickness of trabeculae, besides loss of connectivity between trabeculae, resulting in decreased bone strength.

Osteoporosis is one of the most important age related diseases, and may lead the elderly to physical dependence, increased morbidity and high cost for treatment of the disease. Due to the magnitude of the problem it is important to prevent, through effective programs in reducing the incidence of a major public health problem.^1^

Menopause occurs in most women around the age of fifty. During this period the end of ovulatory function occurs, with decreased estrogen production. The faster the ovarian failure, the higher the possibility of installing severe menopausal syndrome, which is well characterized after surgical castration.²

One of the significant consequences of menopause recognized worldwide is the fact that one in four women aged 65 and older (about 15 years after occurrence of menopause), becomes physically and psychologically invalid due to casualties caused by osteoporosis. Bone loss tends to be more pronounced in the years following menopause or bilateral oophorectomy. Loss of ovarian function is itself a risk for developing osteoporosis. During climacteric the conservation of bone mass should be a concern.^3^

In the literature there are several studies that report that estrogen is the most important hormone in maintaining bone mass and a deficiency of this hormone can be considered a major cause of bone loss related to age in both genders.^4^

Several authors have used bilateral oophorectomy to induce osteoporosis in rats at different time points to examine changes in bone mass through biomechanical and histomorphometric studies.^5^^,^ 6

One must consider, however, the necessity of clinical, biomechanical and histological studies that aim to study jointly the effects of hormone deprivation and natural aging, frequent occurring situation in clinical practice, and for this purpose, it would be more appropriate to adopt longer observation periods covering most of the life cycle of the experimentation animal chosen.

Osteoporosis will be a public health issue in the coming years and the knowledge derived from experimental investigations will certainly contribute significantly, especially in the study of strategies to maintain bone strength in women after menopause, with consequent decrease in the incidence of fractures and associated complications.

## MATERIALS AND METHODS

Sixty four adult virgin female rats, *Rattus norvegicus Albinus*, of the *Wistar* lineage were used, divided into two groups of 32 animals each by random selection: group A (oophorectomy) and group B (placebo). The animals underwent bilateral ovariectomy (group A) and placebo surgery (group B) after reaching the ripening stage, at four months old.

The experiment was conducted at six time points: adaptation; placebo surgery or ovariectomy, named point zero (animals at four months old); sacrifice after three months - point one (seven months old); sacrifice after six months - point two (ten months old); sacrifice after nine months - point three (13 months old); sacrifice after twelve months - point four (16 months old). Both groups A and B were sacrificed simultaneously.

The parameters analyzed were: clinical study: body weight obtained monthly and at times of sacrifice from both groups (placebo and oophorectomy); biomechanical study: mechanical properties (maximum load and stiffness coefficient) and histological study: histomorphometry (trabecular area).

The right tibia of each animal was used to study the biomechanical properties. Tibiae were disarticulated at the proximal femur-tibial region (knee) and at the distal tibial-tarsal articulation (ankle). Then, tibia was removed from the fibula (distally) and all soft tissue (muscles, tendons and ligaments) were removed, leaving only the bone tissue. Then, bones were wrapped in aluminum foil, identified and frozen at -20° C in a domestic freezer. We applied the same technique to the left tibia of each animal used for histological study. Then, the material was fixed in 10% buffered formalin and identified.

For biomechanical studies the Universal Mechanical Testing Machine EMIC*, Model DL 10 000 was used. The system accuracy is plus or minus (0.018 + F/3700) kN calculated within the specifications of ABTN standards, NBR6156 and NBR6674. Bending tests were conducted at three points and the final assay report provided the value of the maximum load (N) and the diagram load-deformation. Then, the value of the proportionality constant (K) or stiffness coefficient (N/mm) was calculated.

**Figure 1:**
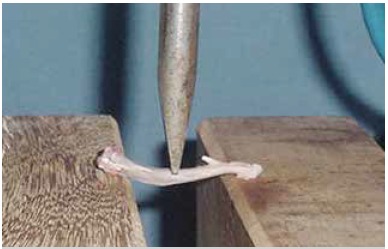
Universal Mechanical Testing Machine with body of proof mounted for three points bending assay.

The specimen was placed with its convex side up, on two supports and the distance between the two support holders was standardized at 2/3 the length of the specimen. The cleaver load application was positioned at a point equidistant to the ends. The load was applied until rupture of the specimen, at a 30 mm/min speed. (Figure 1)

To perform histological study the specimens were prepared and the area of trabecular bone was calculated, next to the epiphyseal plate of the tibia region. The slides were placed on microscope coupled to an image analysis program. The area was calculated in five fields after manual delineation of trabeculae boards. The calculation of the total area was performed automatically by the program.

The study of the following variables: weight, maximum load, and stiffness coefficient was performed by analysis of variance for factorial 2 x 4 (two groups and four sacrifice moments) technique in a randomized model supplemented with Tukey's test for multiple comparisons.^3^

## RESULTS

In the operated group (oophorectomy) the animals significantly gained weight throughout the experiment, reaching the highest average at nine months postoperatively.

Table 1 presents the mean values and standard deviation of body weight according to group and moment of sacrifice, accompanied by the results of the statistical analysis.

**Table 1:** Mean and standard deviation of body weight (g) according to group and time of sacrifice and statistical analysis.

	**M**_1_ ** (3 months)**	**M**_2_ ** (6 months)**	**M**_3_ ** (9 months)**	**M**_4_ ** (12 months)**
Oophorectomy	333 ± 20 aA	396 ± 27 aAB	412 ± 57aB	395 ± 58 aAB
Placebo	343 ± 30 aA	367 ± 48 aA	368 ± 48aA	385 ± 63 aA

DHS (5%) = 53 (for comparison of groups at a given time of sacrifice). DHS (5%) = 71 (for comparison of times of sacrifice within the group). Lowercase: vertical comparison. Capitalization: horizontal comparison. Equal letters: similarity; different letters: significant difference.

Table 2 presents the mean and standard deviation of the variable maximum load according to group and time of sacrifice, accompanied by the results of the statistical analysis.

**Table 2:** Mean and standard deviation of maximum load (N) according to group and time of sacrifice and statistical analysis.

**Group**	**Time of sacrifice**			
	**M**_1_ ** (3 months)**	**M**_2_ ** (6 months)**	**M**_3_ ** (9 months)**	**M**_4_ ** (12 months)**
Operated	63,6 ± 5,3 aA	63,3 ± 14,0 aA	66,6 ± 6,6 aA	74,3 ± 5,6 aA
Control	67,1 ± 6,7 aA	77,7 ± 17,8 bA	71,2 ± 7,2 aA	76,6 ± 9,1 aA

DHS (5%) = 11.5 (for comparison of groups at a given time of sacrifice). DHS (5%) = 15.3 (for comparison of times of sacrifice within the group). Lowercase: vertical comparison. Capitalization: horizontal comparison. Equal letters: similarity; different letters: significant difference.

Table 3 presents the mean and standard deviation of the variable stiffness coefficient according to group and time of sacrifice, accompanied by the results of the statistical analysis.

**Table 3:** Mean and standard deviation of stiffness coefficient (N/mm) according to group and time of sacrifice and statistical analysis.

**Group**	**Time of sacrifice**			
	**M**_1_ ** (3 months)**	**M**_2_ ** (6 months)**	**M**_3_ ** (9 months)**	**M**_4_ ** (12 months)**
Operated	56,5 ± 4,7 aA	52,6 ± 5,3 aA	56,9 ± 4,9 aA	71,8 ± 6,5 aB
Control	65,6 ± 6,7 bA	60,7 ± 7,5 bA	62,0 ± 5,3 aA	82,1 ± 7,9 bB

DHS (5%) = 7.2 (for comparison of groups at a given time of sacrifice).DHS (5%) = 9.6 (for comparison of times of sacrifice within the group). Lowercase: vertical comparison. Capitalization: horizontal comparison. Equal letters: similarity; different letters: significant difference.

Table 4 shows the median values and total average half-width of trabecular area according to group and moment of sacrifice, accompanied by the results of the statistical analysis.

**Table 4:** Median and total average half-width of trabecular area (µm2) according to group and time of sacrifice and statistical analysis.

**Group**	**Time of sacrifice**				**Result of statistical test of time of sacrifice**
	**M**_1_ ** (3 months)**	**M2 (6 months)**	**M**_3_ ** (6 months)**	**M**_4_ ** (12 months)**	
Control	557736 ± 124598aB	419505 ± 165282b AB	349475 ± 93218b AB	286523 ± 244994bA	(p<0,05)
Operated	409130 ± 153657aB	409130 ± 153657aB	126836 ± 78744a A	169742 ± 121697a AB	(p<0,05)
Statistical group test	(p>0,05)	(p<o,o5)	(p<0,05)	(p<0,01)	

Lowercase: vertical comparison. Capitalization: horizontal comparison. Equal letters: similarity; different letters: significant difference.

## DISCUSSION

Ovariectomy was chosen as a model for osteoporosis induction due, possibly, to reproducing the characteristics of post menopause observed in humans. The ovariectomy and placebo surgery occurred at four months old, a period where rats are considered adults.

The animals were divided into two experimental groups, placebo and oophorectomy in order to isolate the effect of hormonal deprivation. The choice of time of sacrifice for studying the variables, namely three, six, nine and twelve month aimed to allow the characterization of any changes along the greater part of the life cycle of the animal.

The removal of the ovaries caused weight gain during the experiment, these results are consistent with literature data that show, after menopause, altered lipid profile due to the change, not only of adipose tissue distribution, caused by estrogen deficiency, but also by the accumulation of peripheral fat. Some authors found that weight gain in the oophorectomized group compared to control at different post-surgical periods.^5^^-^ 8

The most important mechanical properties of bone are strength and stiffness. The mechanical test performed experimentally allows observing the behavior of the bone subjected to loading and in situations like osteoporosis, with loss of bone mass, and estimate the risk of fractures.

In this study, no significant change in the maximum load in both groups over time and in comparison between groups in M2 (six months postoperatively), placebo exhibited maximum load higher then the ovariectomy group. The result showed that three months postoperatively were not sufficient for the induction of resistance loss, which occurred at six months, and returned to statistical equality at nine and twelve months. The explanation for these results may be in several factors such as the values of the standard deviations obtained, which are highly variable, probably due to sample characteristics and individual responses to hormone deprivation. Another explanation is that, at nine months and twelve months postoperatively, there has been progressive loss of bone mass as part of the natural aging process. The result can also be associated with the method of bending test. The load application occurred at the midpoint of the diaphyseal region, composed mainly of compact cortical bone.

More likely, however, is that the groups sacrificed at nine months (13 months old) and 12 months post-operative (16 months old) have progressively lost bone mass as part of the natural aging process, the fact that 'equaled' the mechanical behavior, although the castrated group was longer deprived of hormonal. This theory explains only partially the result that should most likely be linked to the test method. Since we used bending test, load was applied to the midpoint of the diaphyseal region, composed mainly of compact cortical bone.

One can, therefore, assume that the compact cortical bone is more stable and resistant to menopause induced changes, even the natural aging process, which would explain the results obtained and the lowest incidence of fractures in the diaphyseal region, even in cases of severe osteoporosis.

Studies investigating mechanical changes caused by aging in rats also obtained similar outcomes^9^^-^ 11 and reinforce the idea that the mechanical profile is associated not only to hormone level, but also to other factors such as age, diet, activity and others, besides the study method used.

Other authors, in different designed investigations on the effects of menopause on the mechanical properties of bone in rats regarding the age of animals, time of observation, bone and anatomical region beyond the test method, reached several conclusions.^11^^-^ 13

The results of the statistical analysis of the stiffness coefficient showed that both groups, placebo and oophorectomy, showed similar trends over time and the values were only significantly higher at 12 months postoperatively. It can be stated that hormone deficiency, thus, caused decreased bone stiffness and the effect of time (aging) was less pronounced.

Kaplan *et al*.^14^ have studied the reduction of bone mass that occurs due to aging and demonstrated the relationship between bone mass and age in cross-section of the femur. Cortical thinning occurred by decrease in bone mass, which may also be associated with decreased amount of water and other changes in biochemical composition.

Other autores^15^ concluded that resistance was similar in the two age groups (young and elderly), but the old bone was more "brittle", characteristics of "fragile" material and, therefore, with altered stiffness. These studies indicate that in addition to the hormonal factor, the aging factor must have contributed to the result obtained in which the maximum charge was similar, but with different stiffness.

The results of this research showed that estrogen deficiency may alter the stiffness coefficient of the diaphyseal portion and also that changing of this mechanical property does not appear to be directly related to the maximum load resistance. These results indicate, once again, the need for standardization of research methods to clarify the mechanical performance of different bone regions suffering from hormonal deprivation and aging. Other authors, doing bending test on femur of ovariectomized rats, found no significant differences in rigidity coefficient in relation to the control group.^8^^,^ 16

The trabecular area was calculated in the metaphysis, proximal tibia, near the epiphyseal plate, due to the predominance of cancellous bone. There was a significant reduction over time in oophorectomy group and comparison between groups hormone deprivation induced menopause caused a decrease of trabecular area after six months of hormone deprivation, considered young adults, suggesting that there was a decrease in bone mass at that time. These results demonstrate the deleterious effect of hormonal deprivation in trabecular bone and also show that even the effect of aging and natural menopause, present in the placebo group, especially in M3 and M4, were not sufficient to generate greater reduction in trabecular area than animals exposed to hormone deprivation for long periods.

Marques and Taveira^5^ also concluded that there is a direct relationship between aging and the effects of menopause on bone tissue. Other authors investigated estrogen deficiency in rats, using histomorphometry as a research method. However, studies vary regarding the choice of animals age, time of assessment, type and location of bone analysis.^7^^,^ 16 Chen *et al.* 17 observed in ovariectomized rats at five months old after two months, decreased in area, thickness and number of trabeculae in different regions of the proximal tibial metaphysis compared to control. These findings are similar to the study of Carvalho and Cliquet,^8^ who observed structural differences in trabecular bone of the femur in oophorectomized and placebo mature rats and after thirty days of ovariectomy, existing trabeculae in the femur of ovariectomized rats were less frequent and had reduced connectivity.

## CONCLUSION

The simultaneous use of clinical, biochemical and histological parameters in the present investigation, allowed concluding that the removal of the ovaries produces systemic changes, characterized by metabolic alterations that caused weight gain and changes in bone tissue associated with alteration of the mechanical profile and reduction of bone mass.

Apparently, the metaphyseal portion consisting of spongy bone and the diaphyseal portion, with predominantly cortical bone, respond differently to hormone deprivation and aging. It is possible to consider that the metaphyseal region undergoes changes way more intensely and earlier than the diaphyseal region in a state of hormonal deprivation.

The conclusions obtained open the prospect for further studies with a proposal that includes mechanical and histological analyzes of the same anatomical region and can, thus, assess the association between changes and architecture and the corresponding mechanical profile.
